# SG-APSIC1045: A quantitative assessment of ATP bioluminescence on dental instruments reprocessed by automated washer-disinfector and ultrasonic machine

**DOI:** 10.1017/ash.2023.96

**Published:** 2023-03-16

**Authors:** Vivian Man, Tian Cheng Neo

**Affiliations:** National University Polyclinic, Singapore; Dental Services, National University Polyclinics, Singapore

## Abstract

**Objectives:** Dental instruments are contaminated by blood and saliva during dental procedures. To prevent cross infection, all contaminants should be removed from the surfaces of instruments. Inadequate cleaning can hinder disinfection and sterilization process. To compare the cleaning efficacy of an automated washer–disinfector versus an ultrasonic machine on dental instruments, adenosine triphosphate (ATP) measurements were compared. **Methods:** From National University Polyclinic Bukit Panjang Dental Services, we collected 2 loads of 40 dental instruments previously used in dental treatments: extraction forceps, high-volume suction tips, Coupland elevators, matrix band holders, and ultrasonic scaler tips. At the point of use, gross soil was wiped from instrument surfaces with water. Each instrument was swabbed after cleaning either using a washer–disinfector or an ultrasonic machine. The relative light units (RLU) on the luminometer indicated the amount of ATP contaminants and residue bioburden present on the instruments. **Results:** The mean RLU values across all instruments in the washer–disinfector group was 2.5 times lower than the mean value of the instruments in the ultrasonic group (35.4 vs 89.9 RLU). This difference was statistically significant for all instrument groups except for the high-volume suction tips. The Mann-Whitney *U* test indicated that the RLU in the ultrasonic group was higher than the RLU for the washer–disinfector group for extraction forceps (*P* < .001), ultrasonic scaler tips (*P* < .023), and matrix bands (*P* < .006). A *t* test indicated the same relationship for Coupland elevators (*P* < .005). **Conclusions:** The mean RLU values for both cleaning methods were lower than the manufacturer’s benchmark (RLU ≤ 150), suggesting that both methods can achieve effective cleaning. However, cleaning using an automated washer–disinfector is significantly more effective than an ultrasonic machine for nonlumen instruments. The effectiveness of cleaning using ultrasonic machine varied greatly among different types of instruments with different design complexities.

